# When Tachycardia Tells Two Stories: The Diagnostic Dilemma of Concurrent Upper Gastrointestinal Hemorrhage and Alcohol Withdrawal Syndrome in a Post-Coronary Artery Bypass Graft Patient

**DOI:** 10.7759/cureus.113598

**Published:** 2026-07-29

**Authors:** Ann Tanyous, Iten Tanyous, Alaa-Salah Eldin

**Affiliations:** 1 Internal Medicine, Touro College of Osteopathic Medicine, Middletown, USA; 2 Gastroenterology and Hepatology, Hudson Regional Hospital, Secaucus, USA

**Keywords:** alcohol withdrawal syndrome, aspirin gastropathy, coronary artery bypass graft (cabg), glasgow-blatchford score, proton pump inhibitor, sinus tachycardia, upper gastrointestinal bleed

## Abstract

Acute upper gastrointestinal bleeding (UGIB) and alcohol withdrawal syndrome (AWS) are two of the most common medical emergencies encountered in the intensive care unit (ICU). Both conditions share tachycardia as a cardinal sign, yet the clinical implications are diametrically opposed: in UGIB, tachycardia signals hemorrhagic hypovolemia requiring volume resuscitation, while in AWS, tachycardia reflects sympathetic hyperactivity requiring sedation. We present the case of a 57-year-old man with a history of triple coronary artery bypass grafting (CABG) and peptic ulcer disease who presented with hematemesis while on low-dose aspirin without gastroprotective therapy. Esophagogastroduodenoscopy revealed diffuse erythematous gastritis with patchy erosive mucosal changes without active bleeding or varices. Hours after ICU admission, the patient developed tachycardia and tremors, raising the critical question of whether these signs represented rebleeding, emerging alcohol withdrawal, or both. The Clinical Institute Withdrawal Assessment for Alcohol-Revised (CIWA-Ar) scale, the most widely used tool for monitoring AWS, is not validated in patients with concurrent acute medical illness, and its scores can be confounded by coexisting conditions. This case highlights the limitations of standard assessment tools when two life-threatening conditions share overlapping clinical features and proposes a clinical framework for differentiating hemorrhagic tachycardia from withdrawal tachycardia at the bedside. Longitudinal review of the patient's medical records revealed a 15-year progression from denied alcohol use to physiologic dependence, underscoring the importance of serial screening for alcohol use disorder.

## Introduction

Acute upper gastrointestinal bleeding (UGIB) is one of the most common gastrointestinal emergencies, with an estimated incidence of 47-160 per 100,000 persons per year and an in-hospital mortality of approximately 2%-10% [1,2]. Peptic ulcer disease remains the most frequent etiology, although erosive gastritis is a significant contributor, particularly in the setting of aspirin and nonsteroidal anti-inflammatory drug use [3,4]. Low-dose aspirin increases the risk of upper gastrointestinal bleeding by two- to fourfold, with the risk further amplified by prior peptic ulcers, advanced age, *Helicobacter pylori* infection, and concomitant alcohol use [5,6].

Alcohol withdrawal syndrome affects an estimated 2%-7% of patients with heavy alcohol use admitted to the hospital and is characterized by autonomic hyperactivity, including tachycardia, hypertension, tremor, diaphoresis, and, in severe cases, seizures and delirium tremens [7,8]. The Clinical Institute Withdrawal Assessment for Alcohol-Revised (CIWA-Ar) is the most widely used tool for monitoring alcohol withdrawal severity, yet it was originally designed for voluntary detoxification centers and is not validated in critically ill or medically complex patients [9]. The CIWA-Ar relies heavily on subjective patient self-reports for items such as nausea, anxiety, tactile auditory, and visual disturbances, making it particularly susceptible to confounding in patients with concurrent acute medical illnesses [8,9]. The 2020 American Society of Addiction Medicine clinical practice guideline explicitly warns that CIWA-Ar scores can be "falsely elevated due to comorbid conditions (e.g., fever from infection, concurrent withdrawal from another substance) and falsely suppressed due to the use of certain medications (e.g., beta-blockers)" [8].

The concurrent presentation of upper gastrointestinal bleeding and alcohol withdrawal syndrome creates a unique clinical challenge that has received limited attention in the literature. Both conditions produce tachycardia, yet the therapeutic response to each is fundamentally different: volume resuscitation for hemorrhage versus sedation for withdrawal. In UGIB, tachycardia signals hemorrhagic hypovolemia requiring fluid resuscitation and possible transfusion, whereas in AWS, tachycardia reflects sympathetic hyperactivity requiring sedation with benzodiazepines. Critically, the hemodynamic profiles differ: hemorrhagic tachycardia is typically accompanied by hypotension, pallor, and cool extremities, while withdrawal tachycardia is characteristically accompanied by hypertension, diaphoresis, tremor, and agitation [7,8]. Misattribution of tachycardia to the wrong etiology carries life-threatening consequences: excessive sedation in a rebleeding patient may mask hemorrhagic shock, while withholding sedation in a withdrawing patient risks seizures and delirium tremens [5,7,8]. This case report illustrates this diagnostic dilemma and proposes a clinical framework for differentiating the two etiologies of tachycardia when they coexist.

## Case presentation

A 57-year-old man presented to the emergency department with three episodes of hematemesis over approximately 12 hours.

Vital signs on arrival revealed tachycardia (heart rate: 105 beats per minute), blood pressure 124/81 mmHg, respiratory rate 16, temperature 97.2°F, and oxygen saturation 100%. The relatively preserved blood pressure (124/81 mmHg) despite tachycardia (heart rate: 105 beats per minute) may have been partially attributable to the patient's home metoprolol 25 mg twice daily, which can blunt the full tachycardic and hemodynamic response to hypovolemia [8]. The patient appeared comfortable and in no acute distress. The abdomen was soft, nontender, and distended with normal bowel sounds. Skin was warm, dry, and intact without pallor or diaphoresis.

His history included triple coronary artery bypass grafting (CABG) performed eight years prior, hypertension and hyperlipidemia (both diagnosed approximately 12 years prior and on continuous treatment), gout (diagnosed 5 years prior), diverticulitis, and peptic ulcer disease diagnosed about 10 years prior, for which he had been managed intermittently with proton pump inhibitors but was not on gastroprotective therapy at the time of presentation. Additional risk factors for bleeding included advanced age, alcohol use, and the absence of *Helicobacter pylori* testing. The patient had no known history of diabetes mellitus. He had a documented allergy to celecoxib causing "stroke-like symptoms." Celecoxib carries a United States Food and Drug Administration boxed warning for cardiovascular thrombotic events and is contraindicated after coronary artery bypass graft surgery [10,11].

Other regular medications include metoprolol 25 mg twice daily, gabapentin 100 mg three times daily, losartan 50 mg daily, allopurinol 100 mg daily, amlodipine 2.5 mg daily, atorvastatin 80 mg daily, calcium with vitamin D twice daily, and aspirin 81 mg daily. The patient was not on a proton pump inhibitor despite having peptic ulcer disease and multiple risk factors for aspirin-associated bleeding, including advanced age, alcohol use, and prior peptic ulcer disease, and the absence of *Helicobacter pylori* testing.

Social history revealed wine consumption of approximately 4-5 standard drinks per week (1 standard drink equals 14 grams of ethanol, equivalent to approximately 5 ounces of wine) and daily cannabis use for neuropathic pain. Previous records from a prior emergency department visit 15 years earlier (at age 42) for cellulitis reported normal liver function tests and a fasting glucose of 85 mg/dL. The patient also denied all alcohol use at that time. The triple coronary artery bypass grafting was performed 7 years after this prior visit (at age 49), confirming that the patient had multiple healthcare encounters over the intervening 15 years during which alcohol use disorder screening could have been performed.

Two days prior to presentation, the patient reported feeling unwell with poor oral intake. On the morning of presentation, he experienced hematemesis upon rising and noted hematochezia. A second episode of hematemesis occurred in the emergency department.

The most clinically significant pertinent positive findings included tachycardia (heart rate: 105 beats per minute), an elevated blood urea nitrogen (BUN) of 32 mg/dL with a stable creatinine of 0.8 mg/dL yielding a blood urea nitrogen-to-creatinine ratio of 40, leukocytosis (white blood cell count: 11.7 K/uL), hyperglycemia (glucose: 170 mg/dL), elevated total bilirubin of 1.8 mg/dL with normal direct bilirubin, elevated anion gap of 21.8 mEq/L (reference range: 3-12 mEq/L), low carbon dioxide of 20 mmol/L, serum lactate of 3.2 mmol/L (reference range: 0.5-2.0 mmol/L), and positive fecal occult blood. The stable creatinine confirmed that the elevated blood urea nitrogen reflected absorption of blood proteins from the gastrointestinal tract rather than renal dysfunction.

Pertinent negative findings included a hemoglobin of 13.9 g/dL (hematocrit: 41.7%) (within normal limits but potentially masking acute blood loss due to hemoconcentration from poor oral intake) and negative troponin-I (excluding myocardial ischemia as a cause of tachycardia in this post-coronary artery bypass graft patient). The undetectable alcohol level confirmed the patient was not acutely intoxicated and placed him within the withdrawal window. A chest radiograph showed no acute disease.

The elevated anion gap with low carbon dioxide and elevated lactate suggested high anion gap metabolic acidosis, potentially from tissue hypoperfusion related to the gastrointestinal hemorrhage, starvation ketosis, or early alcoholic ketoacidosis in the setting of poor oral intake. The mildly elevated lactate of 3.2 mmol/L could reflect hemorrhage-related hypoperfusion, the metabolic effects of chronic alcohol use (in which an increased NADH-to-NAD ratio favors conversion of pyruvate to lactate), or a combination of both etiologies. Serial lactate monitoring was initiated to assess the trajectory and guide resuscitation. The new hyperglycemia (170 mg/dL) warranted hemoglobin A1c evaluation.

The Glasgow-Blatchford Bleeding Score was 5 (blood urea nitrogen of 28-70: 4 points, heart rate greater than or equal to 100: 1 point), indicating moderate risk requiring hospital-based intervention [12]. Of note, the Glasgow-Blatchford Bleeding Score includes melena (1 point) and syncope (2 points) as history components but does not include hematemesis as a separate scoring element; this patient did not present with melena or syncope, and therefore, no additional history points were assigned [12].

A comparison of laboratory values from the emergency department visit 15 years prior (baseline) and the current presentation is shown in Table 1.

**Table 1:** Comparison of selected laboratory values from a prior emergency department visit 15 years earlier (age 42, presenting with cellulitis; serving as baseline) and the current presentation (age 57, presenting with hematemesis) The stable creatinine confirms that the elevated blood urea nitrogen was due to absorption of blood proteins from the upper gastrointestinal tract rather than renal dysfunction. BUN: blood urea nitrogen, WBC: white blood cell count, H: high, UGIB: upper gastrointestinal bleeding, ED: emergency department

Laboratory test	ED visit 15 years prior	Current presentation	Reference range	Clinical significance
Hemoglobin (g/dL)	15.1	13.9	13.5-16.5	Stable; acute bleed may not yet be reflected
Hematocrit (%)	45.3	41.7	38.3-48.6	Mild decrease; hemoconcentration may mask true blood loss
BUN (mg/dL)	15.6	32 (H)	7-20	BUN-to-creatinine ratio of 40 suggests UGIB
Creatinine (mg/dL)	0.8	0.8	0.6-1.3	Stable; confirms elevated BUN is not renal in origin
Glucose (mg/dL)	85	170 (H)	60-100	New hyperglycemia; warrants hemoglobin A1c
WBC (K/uL)	7.0	11.7 (H)	4.0-10.5	Leukocytosis; stress response or early withdrawal
Platelet count (K/uL)	184	232	130-400	Normal; argues against chronic liver disease
Lactate (mmol/L)	Not obtained	3.2 (H)	0.5-2.0	Mildly elevated; may reflect hypoperfusion, starvation, or early alcoholic ketoacidosis

**Figure 1:**
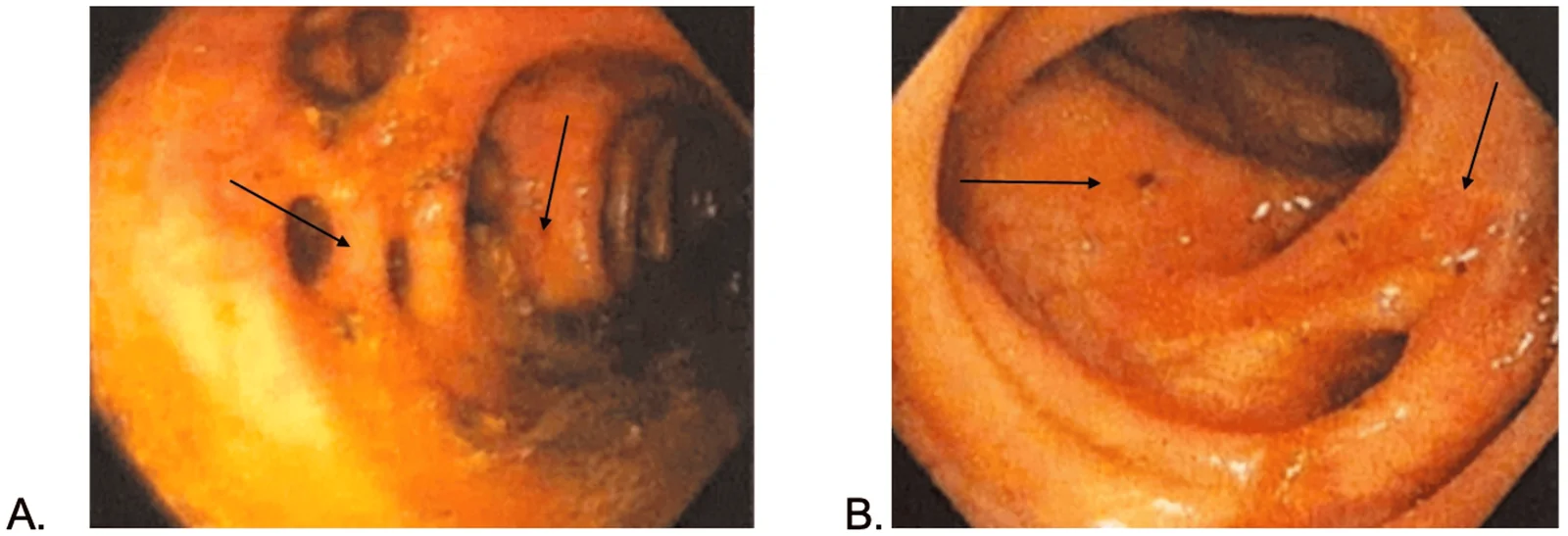
Esophagogastroduodenoscopy findings demonstrating gastritis without active hemorrhage or varices (A) Diffuse erythematous gastritis with patchy erosive mucosal changes (arrows). (B) Gastric lumen with mucosal erythema (arrows) and no esophageal or gastric varices.

Emergent esophagogastroduodenoscopy revealed diffuse erythematous gastritis with patchy erosive mucosal changes (Figure 1A, arrows indicating areas of erosive change) and no esophageal or gastric varices (Figure 1B). No active bleeding was identified. The absence of varices, combined with normal albumin, transaminases, and platelets, confirmed that portal hypertension had not developed despite the alcohol use history. The endoscopic findings were consistent with acid-related mucosal injury in the setting of aspirin use without proton pump inhibitor co-therapy. Management of this non-variceal, non-actively bleeding gastritis consisted of acid suppression with intravenous pantoprazole rather than endoscopic intervention (e.g., clipping or thermal coagulation), which is reserved for lesions with active bleeding or high-risk stigmata such as visible vessels or adherent clots [13]. Had variceal bleeding been identified, management would have included band ligation for esophageal varices or cyanoacrylate injection for gastric varices, along with vasoactive medications (e.g., octreotide) and prophylactic antibiotics [13].

The patient was admitted to the intensive care unit (ICU). Management included pantoprazole 40 mg intravenous bolus followed by 40 mg intravenous push every 12 hours, aspirin held pending confirmed hemostasis, CIWA-Ar monitoring initiated upon ICU admission every 2 hours with chlordiazepoxide 50 mg orally every 2 hours as needed, insulin sliding scale for hyperglycemia, continuation of home antihypertensives and statin, and sequential compression devices for venous thromboembolism prophylaxis [3,9,13]. The clinical team recognized that the CIWA-Ar protocol has known limitations in patients on beta-blocker therapy, as metoprolol can blunt the tachycardic response to withdrawal and falsely suppress CIWA-Ar scores. To account for this confounding effect, the team supplemented CIWA-Ar monitoring with serial hemodynamic assessments, hemoglobin trending every 4-6 hours, and serial lactate measurements. The use of a more objective, non-verbal scoring system such as the modified Minnesota Detoxification Scale (MINDS), which does not rely on patient self-report, was considered but was not available at the institution at the time of this admission [5,8].

Approximately five hours after admission, the patient developed tachycardia (heart rate: 118 beats per minute) and hypertension (blood pressure: 138/86 mmHg) and tremors that were indicative of alcohol withdrawal symptoms rather than electrolyte imbalance or hepatic encephalopathy with asterixis, given the normal electrolytes, normal liver transaminases, and normal albumin. Additional autonomic signs at this time included mild diaphoresis. This constellation of hypertension, tachycardia, diaphoresis, and tremor is characteristic of the symptomatic hyperactivity of alcohol withdrawal, in contrast to hemorrhagic shock, which would be expected to produce hypotension, pallor, cool and clammy skin, and tachycardia without hypertension [7,8]. The attending physician administered lorazepam 2 mg intravenously in addition to chlordiazepoxide and considered initiating a dexmedetomidine (Precedex; Pfizer, New York, NY) infusion.

Over the subsequent 48 hours, the patient's CIWA-Ar scores trended downward from a peak of 18 to below 10, and chlordiazepoxide was tapered accordingly. Serial hemoglobin measurements remained stable at 13.5-13.9 g/dL (hematocrit: 40.5%-41.7%), with no further episodes of hematemesis or hematochezia. Serial lactate measurements normalized from 3.2 mmol/L on admission to 1.4 mmol/L at 12 hours and 0.9 mmol/L at 24 hours, consistent with resolution of hypoperfusion following intravenous fluid resuscitation. The patient received a total of 2.5 liters of lactated Ringer's solution over the first 24 hours of ICU admission. The stability of hemoglobin following adequate volume resuscitation confirmed that the initial hemoglobin of 13.9 g/dL was not artificially elevated by hemoconcentration to a clinically significant degree. Tachycardia resolved by hospital day 3. Aspirin 81 mg daily was resumed on hospital day 2 after confirmed hemostasis, and pantoprazole was transitioned from intravenous to 40 mg orally daily as gastroprotective co-therapy. Pantoprazole was a newly prescribed long-term addition to the patient's medication regimen, as the patient had not been on any proton pump inhibitor at the time of presentation. Hemoglobin A1c returned at 6.1%, consistent with prediabetes. The patient was transferred from the intensive care unit to the medical floor on hospital day 3 and discharged on hospital day 5 in stable condition. Discharge medications included pantoprazole 40 mg daily (newly added as gastroprotective co-therapy), aspirin 81 mg daily, and all prior home medications (metoprolol, gabapentin, losartan, allopurinol, amlodipine, atorvastatin, and calcium with vitamin D). Discharge planning included referral to outpatient gastroenterology for *Helicobacter pylori* testing, referral to addiction medicine for alcohol use disorder evaluation, and a primary care follow-up appointment within one week for glucose monitoring, with repeat hemoglobin A1c planned in three months to confirm the prediabetes diagnosis and assess glycemic trajectory, as a single hemoglobin A1c measurement obtained during acute illness may not fully reflect chronic glycemic status. At the two-week follow-up, the patient reported no recurrent bleeding, was tolerating oral intake, and had reduced alcohol consumption. Repeat complete blood count showed a hemoglobin of 14.2 g/dL, which was consistent with his baseline hemoglobin of 15.1 g/dL from 15 years prior and reflected physiologic recovery following resolution of the acute inflammatory state and restoration of normal erythropoiesis with adequate nutritional intake and iron stores, rather than suggesting that significant blood loss had not occurred.

## Discussion

The central argument of this case is that tachycardia, when it occurs at the intersection of upper gastrointestinal bleeding and alcohol withdrawal, becomes a diagnostically ambiguous sign whose misattribution carries life-threatening consequences. Volume resuscitation for hemorrhage and benzodiazepine sedation for withdrawal are contradictory interventions: excessive sedation in a rebleeding patient may mask hemorrhagic shock, while withholding sedation in a withdrawing patient risks seizures and delirium tremens [5,7,8].

Four features made this distinction particularly challenging. First, the patient's two- to three-day period of poor oral intake placed his last alcohol consumption 48-72 hours prior, squarely within the delirium tremens window [7,8]. Second, metoprolol can suppress the tachycardic response to both hypovolemia and withdrawal, masking severity and falsely suppressing CIWA-Ar scores [8]. Third, the CIWA-Ar is not validated in critically ill patients; the American Association for the Surgery of Trauma consensus document states that "both the CIWA-AR and the BAWS assessments are not validated in critically ill, medically complex, or postoperative patients" [5]. Fourth, the normal hemoglobin may not have reflected true blood loss given hemoconcentration from poor oral intake.

The attending physician's reasoning was instructive: tremors are more specific to alcohol withdrawal than to hemorrhagic shock, which typically produces cool, clammy skin and hypotension rather than tremor. Alcohol withdrawal characteristically produces hypertension, diaphoresis, tremor, and agitation, a constellation of sympathetic hyperactivity that is distinct from the hypotension, pallor, cool extremities, and altered sensorium seen in hemorrhagic shock [7,8]. The blood pressure of 124/81 mmHg on initial presentation, followed by hypertension (138/86 mmHg) at the time of symptom onset five hours later, argued against ongoing hemorrhage and was more consistent with the sympathetic surge of withdrawal. The combination of tachycardia (heart rate: 118 beats per minute), tremors, diaphoresis, hypertension, and a 48- to 72-hour abstinence window was most consistent with emerging withdrawal. Serial hemoglobin measurements every 4-6 hours remain essential in this setting, as a declining hemoglobin should prompt urgent reassessment for rebleeding regardless of concurrent withdrawal symptoms. Objective scales such as the modified Minnesota Detoxification Scale, which does not require patient self-report, may be more appropriate than the CIWA-Ar in critically ill patients with confounding medical conditions [5].

The serum lactate trajectory provided an additional objective parameter for differentiating the two etiologies. The initial lactate of 3.2 mmol/L was mildly elevated and could have reflected either tissue hypoperfusion from hemorrhage or the metabolic effects of chronic alcohol use. However, the rapid normalization to 1.4 mmol/L at 12 hours and 0.9 mmol/L at 24 hours following volume resuscitation argued against ongoing hemorrhagic shock, in which lactate would be expected to remain elevated or rise further. Serial lactate monitoring thus served as a valuable adjunct to serial hemoglobin measurements, providing real-time assessment of tissue perfusion that complemented the slower-trending hemoglobin values.

The blood urea nitrogen-to-creatinine ratio of 40 in this patient was a valuable diagnostic clue. A blood urea nitrogen-to-creatinine ratio greater than 30 carries a likelihood ratio of 7.5 for upper gastrointestinal bleeding [14]. The diagnostic utility of this ratio has been confirmed in a systematic review and meta-analysis, which reported an optimal cut-off of 22 (sensitivity: 66.2%, specificity: 71.0%) [15]. The stable creatinine confirmed that the elevated blood urea nitrogen reflected absorption of blood proteins from the gastrointestinal tract rather than renal dysfunction.

The Glasgow-Blatchford Bleeding Score of 5 placed this patient at moderate risk. This score was originally developed and validated using admission hemoglobin, blood urea, pulse, systolic blood pressure, presentation with syncope or melena, and evidence of hepatic disease or cardiac failure, with a reported receiver operating characteristic curve area of 0.92 [12]. Of note, hematemesis is not a separate scoring component of the Glasgow-Blatchford Bleeding Score; only melena (1 point) and syncope (2 points) are included as history items [12]. The score has been subsequently validated as the best pre-endoscopic risk stratification tool in an international multicenter study, with an area under the receiver operating characteristic curve of 0.90 [14]. Its prognostic utility has been further confirmed in emergency department populations [3].

The endoscopic findings of diffuse erythematous gastritis with patchy erosive mucosal changes are consistent with aspirin-associated erosive gastropathy, which has a point prevalence of approximately 60% in patients on low-dose aspirin [1]. The concurrent presentation of hematochezia alongside hematemesis, despite the absence of active bleeding on esophagogastroduodenoscopy and a stable hemoglobin, is consistent with the known phenomenon of hematochezia occurring in upper gastrointestinal bleeding when brisk or recent hemorrhage results in rapid transit of blood through the gastrointestinal tract before it can be fully degraded to melena. The patient's three episodes of hematemesis prior to endoscopy, combined with the elevated BUN-to-creatinine ratio of 40, confirmed that significant upper gastrointestinal hemorrhage had occurred. The absence of active bleeding at the time of endoscopy suggests that the hemorrhage had ceased spontaneously prior to the procedure, which is consistent with the natural history of erosive gastritis, where bleeding is often self-limited [1,13].

The management of this patient's upper gastrointestinal bleeding followed established protocols for non-variceal UGIB. The endoscopic findings of diffuse gastritis without active bleeding or high-risk stigmata did not warrant endoscopic hemostatic therapy. Acid suppression with intravenous pantoprazole was the appropriate intervention, as proton pump inhibitors facilitate clot formation and stabilization by increasing intragastric pH. Had high-risk stigmata been identified, endoscopic therapy with hemoclips, thermal coagulation, or combination therapy would have been indicated. In contrast, had variceal bleeding been the etiology, management would have required band ligation for esophageal varices or cyanoacrylate injection for gastric varices, along with vasoactive medications and prophylactic antibiotics [13].

The 15-year interval during which alcohol consumption progressed from denied use to physiologic dependence represents a missed preventive opportunity. This progression could not have been detected by the CIWA-Ar, which is a severity assessment tool for active withdrawal rather than a screening instrument for alcohol use disorder. Had validated screening instruments such as the Alcohol Use Disorders Identification Test-Concise been applied at routine clinical encounters, including the multiple healthcare visits for hypertension, hyperlipidemia, gout, and post-CABG follow-up, both the bleeding risk and the withdrawal risk might have been mitigated [6].

The consideration of dexmedetomidine in this patient was particularly strategic. The American Society of Addiction Medicine recommends dexmedetomidine as an adjunct to benzodiazepines (not monotherapy) for alcohol withdrawal-related autonomic hyperactivity [8]. A randomized, double-blind, placebo-controlled study demonstrated that adjunctive dexmedetomidine reduces 24-hour benzodiazepine requirements [16]. A systematic review and meta-analysis confirmed that dexmedetomidine improves sedation quality, although it significantly increases bradycardia risk (relative risk: 2.68, 95% confidence interval: 1.79-4.16) [15]. The alpha-2A agonist mechanism targets sympathetic overdrive without respiratory depression, a critical advantage when excessive benzodiazepine sedation could mask hemorrhagic shock [17]. However, the additive bradycardia risk in a post-coronary artery bypass graft patient on metoprolol warranted careful monitoring. The negative chronotropic and dromotropic synergism between alpha-2 agonists and beta-blockers in a revascularized myocardium presents a significant clinical risk for severe bradycardia, sinus arrest, or conduction blocks. In this patient population, mandatory safety measures include continuous telemetry monitoring, avoidance of loading doses (starting at 0.2 mcg/kg/hour), predefined heart rate thresholds for dose reduction (heart rate less than 55 beats per minute) and infusion discontinuation (heart rate less than 50 beats per minute), and bedside availability of anticholinergic agents (glycopyrrolate or atropine) [10,11,15].

Two modifiable factors contributed to this dual presentation. First, the absence of proton pump inhibitor co-therapy was a critical gap. Proton pump inhibitors have been shown to reduce gastrointestinal events regardless of aspirin dose [18]. The HEAT trial demonstrated that *Helicobacter pylori* eradication reduced peptic ulcer bleeding in aspirin users [19]. This patient, with peptic ulcer disease, alcohol use, and daily aspirin, was an ideal candidate for both interventions [5,18,19]. Second, men consuming more than 30 grams per day of alcohol have a 43% increased relative risk of major gastrointestinal bleeding [3]. Alcohol consumption is an independent risk factor for non-variceal upper gastrointestinal bleeding [20].

Aspirin management in this case followed guideline-concordant practice. Aspirin is recommended indefinitely after coronary artery bypass graft surgery [3]. Current guidelines recommend that aspirin for secondary prevention should be rapidly resumed on the day hemostasis is endoscopically confirmed [3]. The absence of active bleeding on esophagogastroduodenoscopy supported prompt resumption.

## Conclusions

This case illustrates the diagnostic and therapeutic complexity that arises when acute upper gastrointestinal hemorrhage and alcohol withdrawal syndrome coexist. Tachycardia, the most commonly monitored vital sign in both conditions, can represent two fundamentally different pathophysiologic processes requiring contradictory interventions. The CIWA-Ar scale is not validated in patients with concurrent acute medical illness, and clinicians must rely on a synthesis of clinical features, hemodynamic trends, serial lactate measurements, and laboratory trajectories to differentiate the two etiologies. A proposed clinical framework for bedside differentiation includes the following: (1) assessment of accompanying autonomic signs (hypertension, diaphoresis, and tremor favor withdrawal, while hypotension, pallor, and cool extremities favor hemorrhage); (2) serial hemoglobin and lactate trending (declining hemoglobin or rising lactate suggests ongoing hemorrhage, while normalizing lactate with stable hemoglobin argues against active bleeding); (3) temporal correlation with the abstinence window (symptom onset 48-72 hours after last alcohol use favors withdrawal); and (4) response to targeted intervention (improvement with benzodiazepines supports withdrawal, while persistent tachycardia despite sedation should prompt reassessment for hemorrhage).

This case further underscores three preventive opportunities: proton pump inhibitor co-prescription in high-risk aspirin users, *Helicobacter pylori* testing in patients on long-term aspirin therapy, and serial alcohol use disorder screening using validated instruments such as the Alcohol Use Disorders Identification Test-Concise to identify the progressive trajectory of dependence before it manifests as a life-threatening withdrawal syndrome during hospitalization for an unrelated emergency.

## References

[1] Hwang JH, Fisher DA, Ben-Menachem T (2012). The role of endoscopy in the management of acute non-variceal upper GI bleeding. Gastrointest Endosc.

[2] Mueller SW, Preslaski CR, Kiser TH, Fish DN, Lavelle JC, Malkoski SP, MacLaren R (2014). A randomized, double-blind, placebo-controlled dose range study of dexmedetomidine as adjunctive therapy for alcohol withdrawal. Crit Care Med.

[3] Tiglao SM, Meisenheimer ES, Oh RC (2021). Alcohol withdrawal syndrome: outpatient management. Am Fam Physician.

[4] Gauer RL, Abellada A, Stewart M, Kozloski R (2024). Managing selected chronic conditions in hospitalized patients. Am Fam Physician.

[5] (2020). The ASAM Clinical Practice Guideline on Alcohol Withdrawal Management. https://www.asam.org/docs/default-source/quality-science/the_asam_clinical_practice_guideline_on_alcohol-1.pdf.

[6] Cannon JW (2018). Hemorrhagic shock. N Engl J Med.

[7] Darrouj J, Karma L, Arora R (2009). Cardiovascular manifestations of sedatives and analgesics in the critical care unit. Am J Ther.

[8] Aquarius M, Smeets FG, Konijn HW (2015). Prospective multicenter validation of the Glasgow Blatchford bleeding score in the management of patients with upper gastrointestinal hemorrhage presenting at an emergency department. Eur J Gastroenterol Hepatol.

[9] Almadi MA, Lu Y, Alali AA, Barkun AN (2024). Peptic ulcer disease. Lancet.

[10] Aaron AE, Amabile A, Andolfi C (2021). Gastrointestinal Surgical Emergencies Textbook. https://www.scirp.org/reference/referencespapers?referenceid=4203297.

[11] Lau JY, Barkun A, Fan DM, Kuipers EJ, Yang YS, Chan FK (2013). Challenges in the management of acute peptic ulcer bleeding. Lancet.

[12] (2002). Practice guidelines for sedation and analgesia by non-anesthesiologists. Anesthesiology.

[13] Stanley AJ, Ashley D, Dalton HR (2009). Outpatient management of patients with low-risk upper-gastrointestinal haemorrhage: multicentre validation and prospective evaluation. Lancet.

[14] Blatchford O, Murray WR, Blatchford M (2000). A risk score to predict need for treatment for upper-gastrointestinal haemorrhage. Lancet.

[15] Schuckit MA (2014). Recognition and management of withdrawal delirium (delirium tremens). N Engl J Med.

[16] Haber PS (2025). Identification and treatment of alcohol use disorder. N Engl J Med.

[17] Srirajaskanthan R, Conn R, Bulwer C, Irving P (2010). The Glasgow Blatchford scoring system enables accurate risk stratification of patients with upper gastrointestinal haemorrhage. Int J Clin Pract.

[18] FDA Orange Book. (2026). Orange Book: Approved Drug Products with Therapeutic Equivalence Evaluations. https://www.fda.gov/drugs/drug-approvals-and-databases/approved-drug-products-therapeutic-equivalence-evaluations-orange-book.

[19] Wood E, Albarqouni L, Tkachuk S (2018). Will this hospitalized patient develop severe alcohol withdrawal syndrome?: the Rational Clinical Examination systematic review. JAMA.

[20] Díaz LA, König D, Weber S (2025). Management of alcohol use disorder: a gastroenterology and hepatology-focused perspective. Lancet Gastroenterol Hepatol.

